# Modulating *AtDREB1C* Expression Improves Drought Tolerance in *Salvia miltiorrhiza*

**DOI:** 10.3389/fpls.2017.00052

**Published:** 2017-01-24

**Authors:** Tao Wei, Kejun Deng, Qingxia Zhang, Yonghong Gao, Yu Liu, Meiling Yang, Lipeng Zhang, Xuelian Zheng, Chunguo Wang, Zhiwei Liu, Chengbin Chen, Yong Zhang

**Affiliations:** ^1^College of Life Sciences, Nankai UniversityTianjin, China; ^2^School of Life Sciences and Technology, University of Electronic Science and Technology of ChinaChengdu, China; ^3^Center for Informational Biology, University of Electronic Science and Technology of ChinaChengdu, China

**Keywords:** antioxidative metabolism, *AtDREB1C*, drought, photosynthetic capacity, *Salvia miltiorrhiza*, transcriptome

## Abstract

Dehydration responsive element binding proteins are transcription factors of the plant-specific AP2 family, many of which contribute to abiotic stress responses in several plant species. We investigated the possibility of increasing drought tolerance in the traditional Chinese medicinal herb, *Salvia miltiorrhiza*, through modulating the transcriptional regulation of *AtDREB1C* in transgenic plants under the control of a constitutive (35S) or drought-inducible (RD29A) promoter. AtDREB1C transgenic *S. miltiorrhiza* plants showed increased survival under severe drought conditions compared to the non-transgenic wild-type (WT) control. However, transgenic plants with constitutive overexpression of *AtDREB1C* showed considerable dwarfing relative to WT. Physiological tests suggested that the higher chlorophyll content, photosynthetic capacity, and superoxide dismutase, peroxidase, and catalase activity in the transgenic plants enhanced plant drought stress resistance compared to WT. Transcriptome analysis of *S. miltiorrhiza* following drought stress identified a number of differentially expressed genes (DEGs) between the AtDREB1C transgenic lines and WT. These DEGs are involved in photosynthesis, plant hormone signal transduction, phenylpropanoid biosynthesis, ribosome, starch and sucrose metabolism, and other metabolic pathways. The modified pathways involved in plant hormone signaling are thought to be one of the main causes of the increased drought tolerance of AtDREB1C transgenic *S. miltiorrhiza* plants.

## Introduction

Drought is one of the primary abiotic stresses limiting global agricultural production (Zeppel et al., 2014; Shavrukov et al., 2015; Wang et al., 2016). Plant genetic engineering provides an important approach for improving drought resistance in crops via the introduction of transgene(s) to mitigate physiological and agronomic penalties associated with drought (Bihmidine et al., 2013; Patel et al., 2015; Mendiondo et al., 2016). One strategy for increasing drought tolerance is to genetically engineer plants with transcription factors (TF) that control the expression of several abiotic stress response genes (Cabello et al., 2014; de Paiva Rolla et al., 2014; Rehman and Mahmood, 2015). TFs, which are important regulators of gene transcription in various organisms, are classified into a number of families based on the sequences and structures of their DNA-binding domains (Lata and Prasad, 2011; Yamasaki et al., 2013). For example, the plant-specific APETALA2 (AP2)/ethylene-responsive element–binding protein (EREBP) family comprises five subfamilies: AP2, RAV, DREB, ERF, and others (Lata and Prasad, 2011; Licausi et al., 2013; Vahala et al., 2013). DREB TFs, some of the earliest TFs found to be involved in abiotic stress, comprise 56 members divided into six groups (A-1 to A-6) (Hichri et al., 2015). All DREB TFs, which contain a single AP2 domain, bind to the consensus sequence A/GCCGAC in the C repeat or dehydration response element (DRE) in the promoters of genes that are induced under low temperatures and/or water stress (Kovalchuk et al., 2013; Licausi et al., 2013; Dou et al., 2015; Park et al., 2015; Jia et al., 2016). Overexpressing DREB TFs in transgenic plants markedly improves plant survival under both drought and low-temperature conditions (Shan et al., 2007; Byun et al., 2015; Kidokoro et al., 2015; Shavrukov et al., 2015).

When designing genetic engineering strategies to combat abiotic stress in plants, the timing and developmental regulation of the targeted transgene(s) must be coordinated with the occurrence of the corresponding stress (Bihmidine et al., 2013). Misexpression of the targeted transgene(s) in the absence of stress may negatively affect crop performance (Kovalchuk et al., 2013; Shavrukov et al., 2015). For instance, ectopic expression of CBF/DREB genes via fusion to the constitutive 35S CaMV promoter in Arabidopsis (Yamasaki and Randall, 2016), tobacco (Zhou et al., 2014), potato (Storani et al., 2015), and tomato (Li et al., 2012) improved plant performance under abiotic stress but resulted in developmental abnormalities that, under non-stress environments, penalized plant performance. Therefore, precise regulation of the timing and expression level of the transgene is an important consideration when designing constructs to be introduced into a plant genome. For example, when stress-inducible promoters were used in transgenic cassettes carrying transgenes thought to improve abiotic stress tolerance in rice (Ravikumar et al., 2014), barley (Kovalchuk et al., 2013), wheat (Morran et al., 2011), and tomato (Rai et al., 2013), phenotypic development was normal in the transgenic lines under non-stress conditions, yet plant performance was still improved under stress conditions.

*Salvia miltiorrhiza*, commonly known as Danshen, is a widely used traditional Chinese medicinal plant; its roots are used to treat hyperlipidemia as well as cardiovascular and cerebrovascular diseases (Cui et al., 2015; Luo et al., 2015; Xu et al., 2015; Zhou et al., 2016). The recent decline in environmental conditions, especially the increased drought conditions in soil due to higher global temperatures, has resulted in a sharp reduction in the planting area and yield of *S. miltiorrhiza* (Wu et al., 2014; Zhang et al., 2016). Therefore, it is important to improve the stress resistance and adaptability of this plant. However, to date, efforts to improve this plant by genetic engineering have mainly focused on increasing the contents of active ingredients such as tanshinone and phenolic acids (Zhang Y. et al., 2014; Zhao et al., 2015). By contrast, few studies have focused on improving the stress tolerance of *S. miltiorrhiza* using transgenic methods (Wang et al., 2012; Wu et al., 2014).

Arabidopsis contains three DREB1/CBF genes in tandem on chromosome 4 in the order *DREB1B/CBF1*, *DREB1A/CBF3*, and *DREB1C/CBF2* (Park et al., 2015; Jia et al., 2016). The high level of similarity among these genes, as well as their tandem organization and the observation that they have the same transcriptional orientation, suggest that share a common origin (Kang et al., 2013). Overexpression of AtDREB1A, AtDREB1B, and AtDREB1C in Arabidopsis helped confirm that they have matching functional activities; the phenotypes of these lines mimicked the multiple biochemical changes associated with cold acclimation (Gilmour et al., 2004). We previously demonstrated that the drought resistance of both AtDREB1A and AtDREB1B transgenic *S. miltiorrhiza* plants was significantly improved under severe drought stress (Wei et al., 2016a,b). However, the effects of AtDREB1C overexpression in transgenic *S. miltiorrhiza* has not previously been examined, and whether overexpressing *AtDREB1C* can improve the drought tolerance of transgenic plants remains to be determined.

In this study, we demonstrated that ectopic expression of *Arabidopsis DREB1C* led to substantial improvements in the capacity of *S. miltiorrhiza* to survive under severe drought stress conditions. We assessed the effects of ectopic expression of *AtDREB1C* on drought resistance in *S. miltiorrhiza* by investigating shoot growth, the photosynthetic system (stomatal conductance rate, transpiration rate, and net photosynthesis rate), antioxidative metabolism (both antioxidant enzymes and lipid peroxidation), and transcriptomic changes in transgenic plants. We found that expressing *AtDREB1C* under the control of the stress-inducible RD29A promoter greatly improved drought tolerance and minimized the negative effects of this gene on plant growth, as were observed using the strong constitutive CaMV35S promoter.

## Materials and Methods

### Vector Construction

**FIGURE 1:**
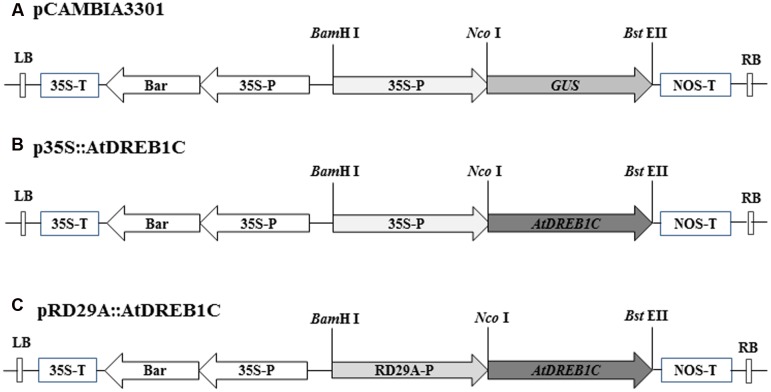
**T-DNA regions of the binary vectors employed for *Agrobacterium tumefaciens*-mediated transformation. (A)** Diagram of binary vector pCAMBIA3301. **(B)** Binary vector p35S::AtDREB1C, containing *AtDREB1C* driven by the 35S promoter. **(C)** Binary vector pRD29A::AtDREB1C, containing *AtDREB1C* driven by the RD29A promoter. 35S-P, cauliflower mosaic virus-CaMV35S RNA promoter; RD29A-P, *Arabidopsis thaliana* RD29A promoter; 35S-T, CaMV35S poly A; NOS-T, 3′ terminator region of the nopaline synthase gene; Bar, phosphinothricin (R); RB, right border; LB, left border.

The full-length open reading frame of *AtDREB1C* (GenBank accession number: AB013817) and the *Arabidopsis thaliana RD29A* promoter region (GenBank accession number: AY973635) were PCR-amplified from genomic DNA using primers containing the restriction sites *Nco*I/*Bst*EII and *Bam*HI/*Nco*I, respectively (Supplementary Table S1). The resulting PCR products were cloned into the pEASY-T1 vector (TransGen Biotech, Beijing, China). Following digestion, the *AtDREB1C* fragment was inserted into binary plant vector pCAMBIA3301, containing the CaMV35S constitutive promoter (**Figure 1**). To construct the pRD29A::AtDREB1C plasmid, the 35S promoter from p35S::AtDREB1C was replaced with the RD29A promoter using *Bam*HI and *Nco*I (**Figure 1**). The vectors were individually transformed into *Agrobacterium tumefaciens* strain LBA4404 using standard heat-shock methods prior to use in the plant transformation experiments.

### Plant Materials, Genetic Transformation, and Molecular Characterization

*Salvia miltiorrhiza* seeds were surface sterilized with 0.1% HgCl_2_ and cultured on hormone-free MS agar medium containing 30 g L^-1^ sucrose and 8 g L^-1^ agar. The cultures were incubated in a growth chamber at 25 ± 2°C under a 16 h light/8 h dark cycle provided by a cool white fluorescent lamp, providing a PPFD level of 26 μmol m^-2^ s^-1^. The *S. miltiorrhiza* plants were transformed via the Agrobacterium-mediated leaf-disk method (Wei et al., 2016b), followed by weekly subculturing of the leaf disks. After 35 days, the shoots were cut off of the plants and transferred into selective rooting medium, leading to the production of roots within 2 weeks. The rooted plantlets were cut into segments from their internodes and propagated on a 1/2-strength MS basal medium; 1.5-month-old plantlets were used for PCR screening and for gene expression evaluations. Positive transformants were transferred to soil for further observation and stress treatments.

The cetyltrimethylammonium bromide (CTAB) method was used to isolate genomic DNA from young leaves (Wei et al., 2016b). Positive pCAMBIA3301 transgenic lines were detected using GUS-F/R. Primer sets used for the p35S::AtDREB1C transgenic lines were designed based on the sequences of the 35S promoter of pCAMBIA3301 (for the forward primer, 35S-F) and *AtDREB1C* (for the reverse primer, AtDREB1C-R). Similarly, RD29A-F/AtDREB1C-R was used to detect pRD29A::AtDREB1C transgenic plants (Supplementary Table S1). Primers 18S-F/R were designed based on the conserved regions of the control gene, 18S rRNA.

A modified CTAB method was used to extract total RNA from WT and transgenic lines after drought stress treatment as described previously (Wei et al., 2016b). Semi-quantitative RT-PCR was performed to investigate *GUS* or *AtDREB1C* expression in transgenic *S. miltiorrhiza* lines using specific primers (Supplementary Table S1). The actin gene was used as an internal control.

### Stress Treatment

Transgenic *S. miltiorrhiza* seedlings were grown in a growth chamber (4°C) for cold treatment. For abscisic acid (ABA), drought, and salt stress treatments, the seedlings were carefully rinsed with water and transferred to 1/4 MS solutions containing 100 μM ABA, 20% PEG6000, or 250 mM NaCl, respectively. Treatment with 1/4 MS solution lacking supplements was used for the control. After 6 h, control and stress-treated seedlings were harvested, immediately frozen in liquid nitrogen, and stored at -80°C for further analysis (Wei et al., 2016a).

To analyze drought tolerance in transgenic *S. miltiorrhiza* plants, plantlets in sterile tubes in solid MS medium were transferred to sterile soil containing nutrients and cultured in a growth chamber at 25 ± 2°C under a 16-h/8-h photoperiod (light intensity, 50 μmol m^-2^ s^-1^). Prior to drought stress treatment, the pots for both the well-watered and drought-stress treatments were saturated with water and allowed to drain overnight. Plants at uniform developmental stages were chosen for stress treatments. On day 54, the plants were stressed by withholding water for 9 days, the relative water content (RWC) and other physiological parameters were measured in the leaves using the third-youngest leaf of three randomly selected plants per treatment. After 11 days of drought stress treatment, since all plants showed symptoms of severe wilting, the plants were watered again.

### Determining Physiological Indices

O_2_^-^ and H_2_O_2_ levels were measured as previously described (Wei et al., 2016a). The RWC and chlorophyll content of both WT and transgenic plants were determined as described (Wei et al., 2016b). Leaf gas exchange parameters, including stomatal conductance (gs), transpiration rate (E), and net photosynthesis rate (Pn) were measured using a portable gas exchange system (CIRAS-2, PP-System, Amesbury, MA, USA) (Wei et al., 2016a). Malondialdehyde (MDA) content and superoxide dismutase (SOD), peroxidase (POD), and catalase (CAT) activity were evaluated as described (Wei et al., 2016a).

### Transcriptome Analysis

Total RNA from the mixed sample (three individual plants) was isolated using the modified CTAB method as described above. Following RNase-free DNase I treatment (New England BioLabs) for 30 min at 37°C to remove residual DNA, the samples were sequenced by Biomarker Technologies Co., Ltd. (Beijing, China), and cDNA library construction and Illumina HiSeq2500 sequencing were performed as described previously (Wei et al., 2016b).

### Validation of RNA-seq Data by qRT-PCR

A total of 30 pairs of gene-specific primers (Supplementary Table S1) were designed to produce amplicons for validating the RNA-seq data. Quantitative reverse-transcription PCR (qRT-PCR) was performed on an iQ5.0 instrument (Bio-Rad, USA) using SYBR Green qPCR kits (Roche) according to the manufacturer’s protocol. Relative gene expression levels were calculated using the 2^-ΔΔCt^ method; expression levels were quantified by normalization against *Actin* and *GAPDH*. All assays for each gene were performed in triplicate synchronously under identical conditions.

### Statistical Analysis

All data are presented as the means ± standard error (SE) of at least three replicates. Statistical analysis (analysis of variance; ANVOA) was performed using SAS software version 9.1 (SAS Institute Inc., Cary, NC, USA) to test for significant differences between WT and transgenic lines. The mean values of each treatment group were compared using Duncan’s test at *P* < 0.05.

## Results

### Generation of Transgenic Lines

**FIGURE 2:**
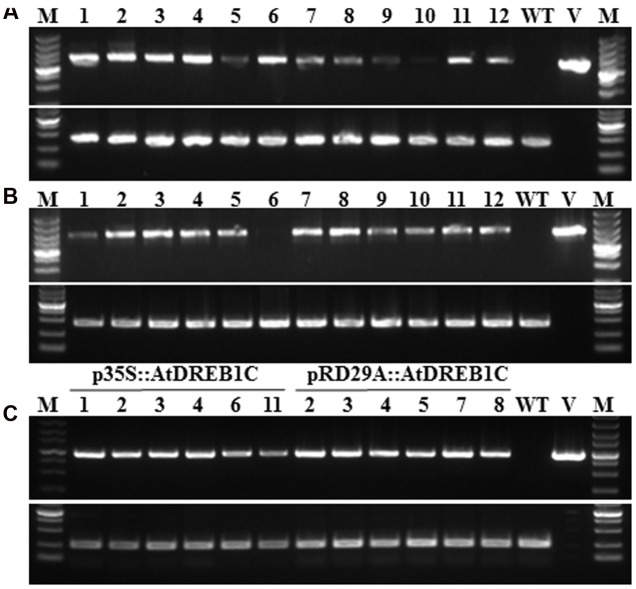
**Identification of transgenic AtDREB1C *Salvia miltiorrhiza* plants via PCR. (A)** PCR analysis of primary transformants using specific primers for *35S::AtDREB1C*. Lanes 1 and 16, size markers; lanes 2–13, DNA from putative transformants; lane 14, untransformed control; lane 15, p35S::AtDREB1C. The 18S rRNA gene was used as a control. **(B)** PCR analysis of primary transformants using specific primers for *RD29A::AtDREB1C*. Lanes 1 and 16, size marker; lanes 2–13, DNA from putative transformants; lane 14, untransformed control; lane 15, pRD29A::AtDREB1C. The 18S rRNA gene was used as a control. **(C)** RT-PCR analysis of *AtDREB1C* expression in transgenic plants using under drought conditions. Lanes 1 and 16, size markers; lanes 2–7, DNA from PCR-positive *p35S::AtDREB1C* transformants; lanes 8–13, DNA from PCR-positive *pRD29A::AtDREB1C* transformants; lane 14, untransformed control; lane 15, p35S::AtDREB1C and pRD29A::AtDREB1C. The actin gene served as the internal control.

To examine the role of *AtDREB1C/CBF2* in *S. miltiorrhiza*, we constructed transgenic plants harboring p35S::AtDREB1C and pRD29A::AtDREB1C (**Figure 1**), expressing *AtDREB1C* under the control of the constitutive CaMV35S and the stress-inducible RD29A promoter, respectively. We selected putative transgenic lines harboring these constructs on medium containing Basta and confirmed them using PCR with gene-specific primers (**Figures 2A,B**). We performed RT-PCR analysis using cDNA from the transgenic plants (p35S::AtDREB1C and pRD29A::AtDREB1C), which amplified the 918 bp *AtDREB1C* fragment, under stress conditions (**Figure 2C**). A 1081 bp PCR product corresponding to the region harboring the *GUS*-specific primer from the pCAMBIA3301 vector control plants was detected (data not shown). We selected two independent p35S::AtDREB1C transgenic lines (p35S::AtDREB1C-01 and 04) and two pRD29A::AtDREB1C transgenic lines (pRD29A::AtDREB1C-02 and 07), which produced high levels of AtDREB1C after drought stress, for further analysis.

### *AtDREB1C* Expression Patterns in Transgenic *S. miltiorrhiza* Plants

**FIGURE 3:**
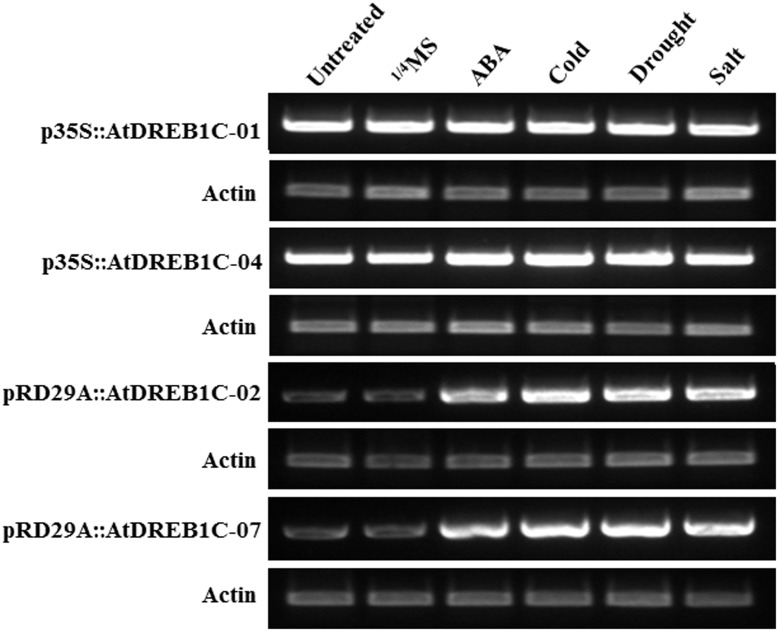
**Induction of *AtDREB1C* expression under various conditions.** ABA- and stress-induced *AtDREB1C* expression determined by RT-PCR. RNA was isolated from seedlings subjected to stress treatments as follows: 100 μM ABA (6 h), 250 mM NaCl (6 h), or 20% PEG6000 (6 h) in 1/4 Murashige and Skoog (MS) solution; cold (4°C, 6 h). Untreated indicates untreated control plants, and 1/4 MS indicates seedlings treated with 1/4 MS solution lacking supplements. The transgenic lines are indicated by the construct names followed by the line number: p35S::AtDREB1C-01 refers to p35S::AtDREB1C line 01 and so on.

We performed RT-PCR to investigate the effects of exogenous ABA treatment and various stress conditions on *AtDREB1C* expression. We detected a relatively high level of *AtDREB1C* expression in the p35S::AtDREB1C transgenic lines (p35S::AtDREB1C-01 and 04) under both untreated and various stress-treated conditions (**Figure 3**). However, *AtDREB1C* was expressed at very low levels in the pRD29A::AtDREB1C transgenic lines (pRD29A::AtDREB1C-02 and 07) without stress, but strongly induced after exogenous ABA application and various stress treatments (**Figure 3**).

### Morphological Changes and Drought Tolerance in Transgenic *S. miltiorrhiza* Plants

**FIGURE 4:**
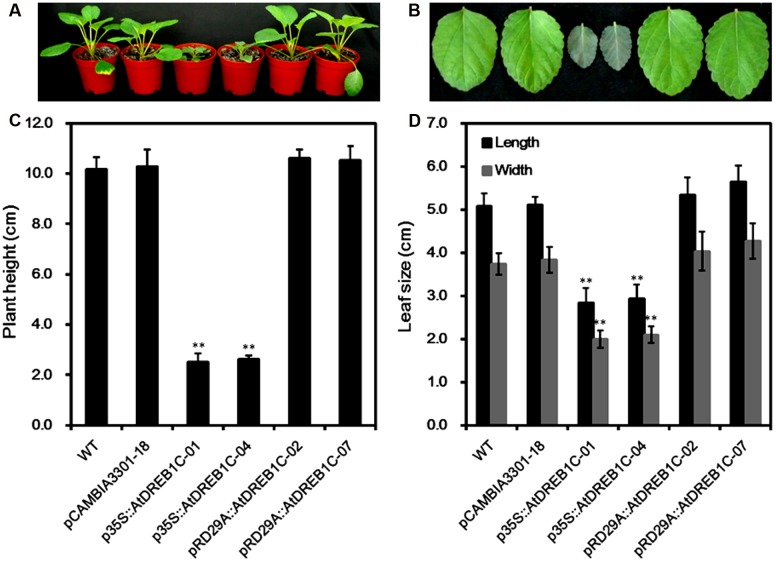
**Growth of AtDREB1C transgenic *S. miltiorrhiza* plants. (A)** Wild-type and transgenic seedlings. **(B)** Wild-type and transgenic leaves. **(C)** Plant height (cm) in wild-type and transgenic lines. **(D)** Leaf size (cm) in wild-type and transgenic lines. The labels under the graphs correspond to the plant materials shown in **(A)** and **(B)**. WT, wild type. The transgenic lines are indicated by the construct names followed by the line number: p35S::AtDREB1C-01 refers to p35S::AtDREB1C line 01 and so on; pCAMBIA3301-18 was used for the positive control. Bars represent the mean + SE of three independent experiments. ^∗∗^Significantly different at the *P* < 0.01 level compared to WT.

We observed the growth of the *AtDREB1C* transgenic *S. miltiorrhiza* plants under normal growing conditions and compared them with WT plants and pCAMBIA3301 vector control plants. Both p35S::AtDREB1C transgenic lines showed significant growth retardation compared to the control plants, whereas the growth of the pRD29A::AtDREB1C transgenic lines was unaffected (**Figure 4A**). The average plant height of the p35S::AtDREB1C transgenic lines was less than 1/3 that of WT plants (**Figure 4C**). In addition, the leaves of p35S::AtDREB1C transgenic plants were darker and shorter than those of WT (**Figure 4B**). The leaf size (length and width) was also smaller in the p35S::AtDREB1C plants than in WT (**Figure 4D**).

**FIGURE 5:**
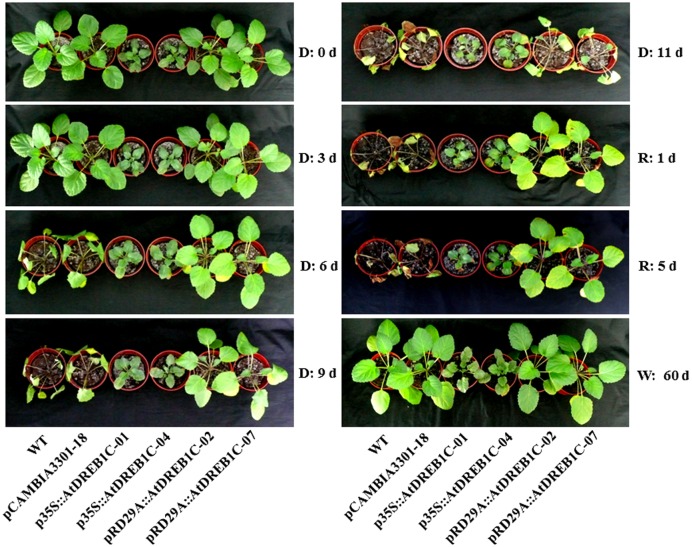
**Morphological changes in wild-type and transgenic plants under drought stress.** D, drought; R, re-watered; W, well-watered; WT, wild type. The transgenic lines are indicated by the construct names followed by the line number: p35S::AtDREB1C-01 refers to p35S::AtDREB1C line 01 and so on; pCAMBIA3301-18 was used for the positive control.

To investigate whether *AtDREB1C* expression driven by the different promoters was associated with increased stress tolerance, we subjected WT and transgenic plantlets to 11 days of drought stress. At the early stage of treatment (3 days), all plants showed normal growth (**Figure 5**). At day 6 of treatment, the leaves of WT and the control line pCAMBIA3301-18 rapidly withered, whereas the growth of p35S::AtDREB1C and pRD29A::AtDREB1C plants was unaffected by drought stress. After 9 days of drought stress, both WT and pCAMBIA3301-18 plants exhibited severe wilting. By contrast, the growth of p35S::AtDREB1C and pRD29A::AtDREB1C transgenic plants was almost normal under drought stress treatment. However, on the last day of drought treatment (day 11), all plants, including p35S::AtDREB1C and pRD29A::AtDREB1C transgenic plants, were severely wilted (**Figure 5**). After re-watering, both p35S::AtDREB1C and pRD29A::AtDREB1C transgenic plants recovered quickly, whereas the growth of drought-stressed WT and pCAMBIA3301-18 plants was severely inhibited; the plants failed to recover and ultimately died (**Figure 5**). All lines, including WT and pCAMBIA3301-18, grew well under well-watered conditions.

### Ectopic Expression of *AtDREB1C* Reduces ROS Accumulation under Drought Stress

**FIGURE 6:**
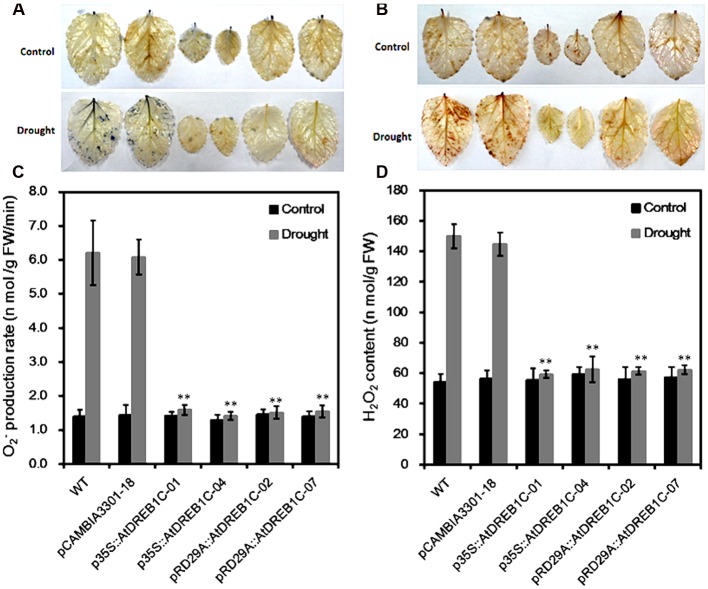
**Changes in O_2_^-^ (A,C)** and H_2_O_2_ **(B,D)** levels in wild-type (WT) and transgenic leaves from plants subjected to drought stress. Drought-stressed leaves were incubated in nitroblue tetrazolium (NBT) or diaminobenzidine (DAB) solution. Blue staining indicates the location and levels of O_2_^-^ **(A)**. Brown staining indicates H_2_O_2_ accumulation **(B)**. O_2_^-^ production rate **(C)** and H_2_O_2_ content **(D)** were measured after drought treatment. The labels under the graphs correspond to the plant materials shown in **(A)** and **(B)**. Control, plants growing under normal conditions; Drought, plants growing after drought stress treatment (6 days). WT, wild type. The transgenic lines are indicated by the construct names followed by the line number: p35S::AtDREB1C-01 refers to p35S::AtDREB1C line 01 and so on; pCAMBIA3301-18 was used for the positive control. Bars represent the mean + SE of three independent experiments. ^∗∗^Significantly different at the *P* < 0.01 level compared to WT.

Since drought stress increases ROS production, we investigated whether transgenic modification would reduce ROS accumulation after drought stress by examining O_2_^-^ and H_2_O_2_ accumulation in the plants using NBT and DAB staining, respectively. Under normal conditions, little NBT staining was detected in the WT and pCAMBIA3301-18, whereas after drought stress, clear NBT staining was detected in both lines (**Figure 6A**). By contrast, little NBT staining was detected in the *AtDREB1C* transgenic lines, even under drought stress. In addition, lower levels of H_2_O_2_ were detected in the transgenic lines than in the WT and vector control lines, as revealed by DAB staining (**Figure 6B**). Quantitative analysis of O_2_^-^ and H_2_O_2_ levels indicated that the transgenic lines produced significantly lower levels of ROS than WT (**Figures 6C,D**).

### *AtDREB1C* Expression Affects MDA Content, as Well as SOD, POD, and CAT Activity

**FIGURE 7:**
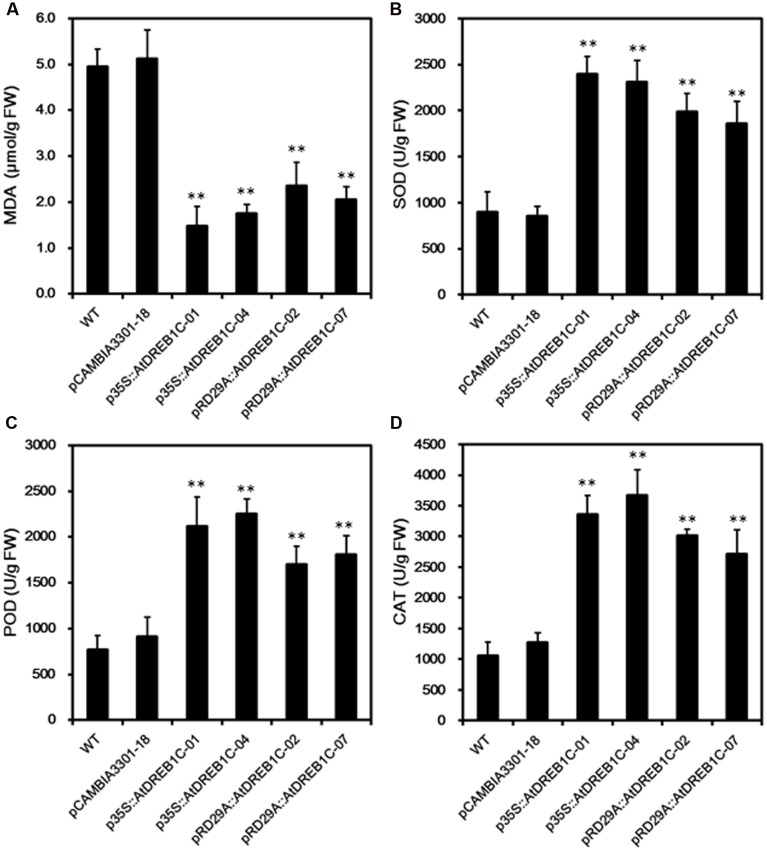
**Malondialdehyde levels (A)** and SOD **(B)**, POD **(C)**, and CAT **(D)** activity in WT and transgenic *S. miltiorrhiza* plants after withholding watering for 9 days. WT, wild type. The transgenic lines are indicated by the construct names followed by the line number: p35S::AtDREB1C-01 refers to p35S::AtDREB1C line 01 and so on; pCAMBIA3301-18 was used for the positive control. Bars represents the mean + SE of three independent experiments. ^∗∗^Significantly different at the *P* < 0.01 level compared to WT.

We measured MDA content, an indicator of lipid peroxidation, in the leaves of WT and transgenic lines. After 9 days of drought treatment, we detected significantly lower MDA levels (*P* < 0.01) in the transgenic lines than in WT and control line pCAMBIA3301-18 (**Figure 7A**). We further analyzed drought stress responses in the plants by monitoring SOD, POD, and CAT activity; these enzymes scavenge harmful ROS that accumulate under stress. The trends in the activities of these antioxidant enzymes were similar in all lines after 9 days of drought stress (**Figures 7B–D**). The activities of all antioxidant enzymes were higher in the AtDREB1C transgenic lines than in WT and pCAMBIA3301-18, with a slight difference observed between p35S::AtDREB1C and pRD29A::AtDREB1C transgenic plants. These findings suggest that the enhanced drought tolerance in the AtDREB1C transgenic lines (both the 35S and RD29A promoter lines) is related to their reduced MDA content and increased antioxidant enzyme activity.

### Overexpressing *AtDREB1C* Increases RWC and Chlorophyll Content in Transgenic *S. miltiorrhiza* Plants

**FIGURE 8:**
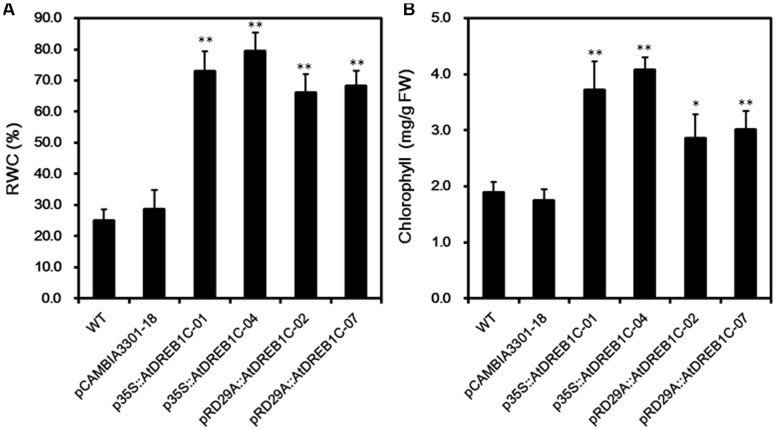
**Effects of drought stress on relative water content (RWC) (A)** and chlorophyll content **(B)** in WT and transgenic *S. miltiorrhiza* plants after withholding watering for 9 days. WT, wild type. The transgenic lines are indicated by the construct names followed by the line number: p35S::AtDREB1C-01 refers to p35S::AtDREB1C line 01 and so on; pCAMBIA3301-18 was used for the positive control. Bars represent the mean + SE of three independent experiments. ^∗^Significantly different at the *P* < 0.05 level compared to WT. ^∗∗^Significantly different at the *P* < 0.01 level compared to WT.

We calculated RWC and chlorophyll content in the plants after 9 days of drought stress. As shown in **Figure 8A**, the RWC was higher in all AtDREB1C transgenic lines than in the WT and vector control line (pCAMBIA3301-18), whereas there was no significant difference in these values between the p35S::AtDREB1C and pRD29A::AtDREB1C transgenic lines. The transgenic lines had significantly higher chlorophyll contents than WT and the vector control (pCAMBIA3301-18; *P* < 0.01), with p35S::AtDREB1C-04 plants showing the highest value (**Figure 8B**).

### Increased Photosynthetic Capacity in AtDREB1C Transgenic Plants under Drought Stress

**FIGURE 9:**
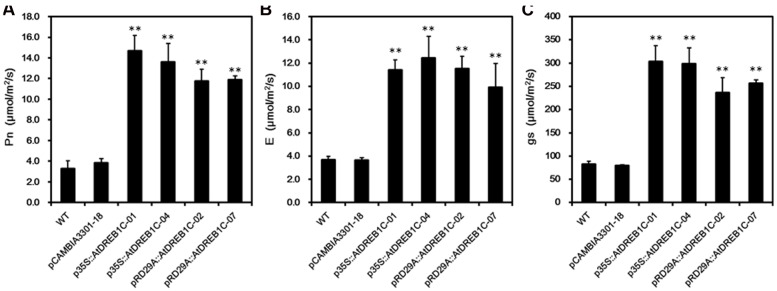
**Effects of drought stress on leaf gas exchange parameters in WT and transgenic *S. miltiorrhiza* plants after withholding watering for 9 days. (A)** Net photosynthesis rate (Pn). **(B)** Transpiration rate (E). **(C)** Stomatal conductance (gs). WT, wild type. The transgenic lines are indicated by the construct names followed by the line number: p35S::AtDREB1C-01 refers to p35S::AtDREB1C line 01 and so on; pCAMBIA3301-18 was used for the positive control. Bars represent the mean + SE of three independent experiments. ^∗∗^Significantly different at the *P* < 0.01 level compared to WT.

To assess the photosynthetic capacity of the transgenic lines, we used a portable photosynthesis system (CIRAS-2) to measure Pn, E, and gs-values over the course of drought treatment in soil. Under well-watered conditions, these values varied little among *S. miltiorrhiza* lines throughout the study period (data not shown). However, when subjected to drought stress, the WT and control line (pCAMBIA3301-18) exhibited a significant decline in Pn, E, and gs, whereas the AtDREB1C lines were less strongly affected by this treatment. The greatest difference was observed on day 9 of drought treatment, with no significant differences detected between the p35S::AtDREB1C and pRD29A::AtDREB1C lines (**Figure 9**).

### Transcriptome Sequencing of AtDREB1C-Overexpressing *S. miltiorrhiza* under Drought Stress

To further investigate the molecular mechanisms underlying the enhanced drought tolerance in the AtDREB1C overexpression lines, we compared the global expression profiles of lines p35S::AtDREB1C-04 and pRD29A::AtDREB1C-07 versus WT following drought treatment. Mixed RNA samples from three separate 51-day-old plants (after 6 days of drought) were used to generate a cDNA library, yielding a total of 18,810,158, 17,445,426, and 18,966,357 clean reads for the WT, p35S::AtDREB1C-04, and pRD29A::AtDREB1C-07 cDNA libraries, respectively. The guanine–cytosine (GC) contents in these libraries were 50.16, 48.50, and 49.77%, respectively, and all Q30 percentages were higher than 85% (Supplementary Table S2). As no reference genome was available, we constructed a *de novo* transcriptome assembly by combining all clean reads from different cDNA libraries. High-throughout sequencing identified 13,153,489 contigs, 161,844 transcripts, and 73,748 unigenes (Supplementary Table S3). To obtain functional annotations, we assigned the unigenes to categories in the COG, GO, KEGG, KOG, Pfam, Swiss-Prot, and NR databases (Supplementary Table S4). A total of 43,428 unigenes were identified, 14,182 of which were enriched in the COG database, while 26,570 were analyzed in the GO database, and 19,338 were mapped to KEGG pathways.

**FIGURE 10:**
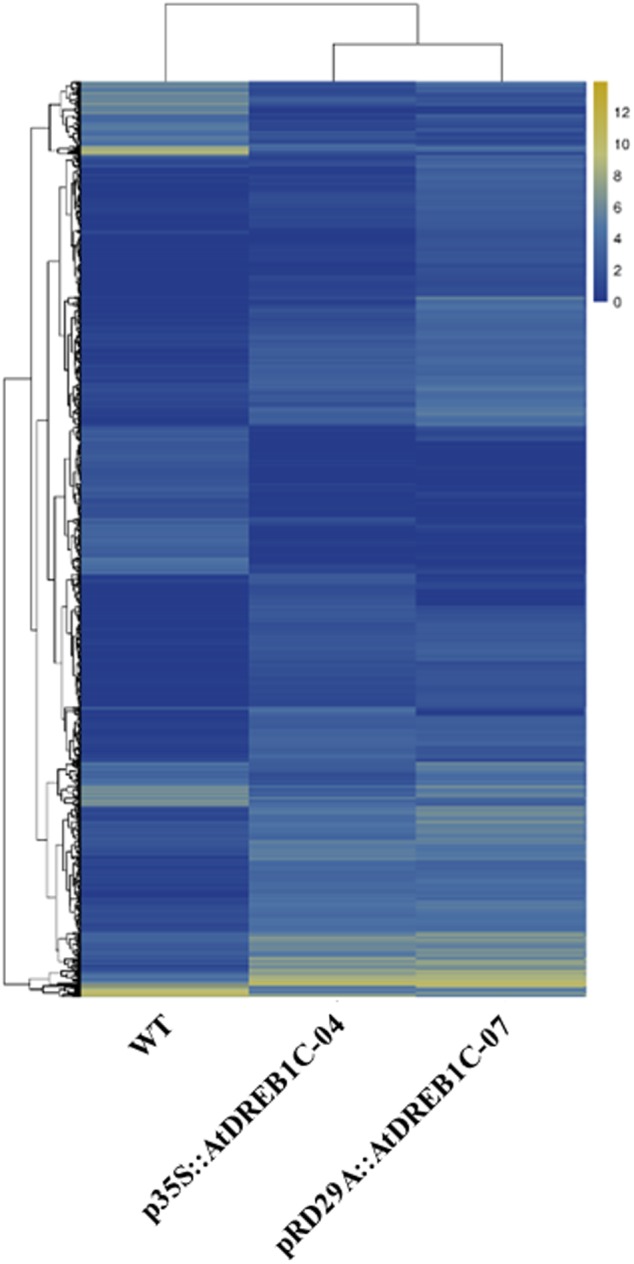
**Clustering analysis of DEGs in WT and AtDREB1C transgenic *S. miltiorrhiza* plants based on their expression profiles obtained by RNA-Seq.** WT, wild type; p35S::AtDREB1C-04, p35S::AtDREB1C transgenic line 04; pRD29A::AtDREB1C-07, pRD29A::AtDREB1C transgenic line 07. The color scale corresponds to the log2 (FPKM) values of genes in various samples.

**FIGURE 11:**
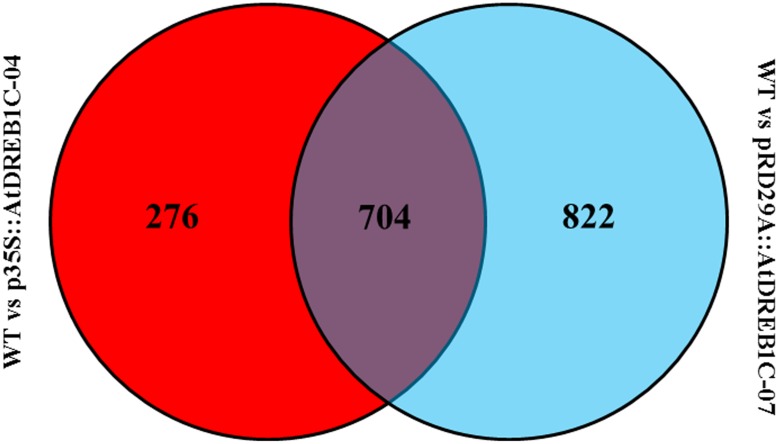
**Venn diagram analysis of DEGs between two comparisons (WT versus p35S::AtDREB1C-04 and WT versus pRD29A::AtDREB1C-07).** WT, wild type; p35S::AtDREB1C-04, p35S::AtDREB1C transgenic line 04; pRD29A::AtDREB1C-07, pRD29A::AtDREB1C transgenic line 07.

We analyzed the expression of the unigenes in the WT and two transgenic lines using Bowtie (Langmead et al., 2009) and RSEM software (Li and Dewey, 2011) and normalized the values by FPKM (Fragments Per Kilobase of transcript per Million mapped reads) (Trapnell et al., 2010). A total of 1,970 differentially expressed genes (DEGs) were identified via clustering analysis (**Figure 10**), 704 of which were same between two comparisons (WT versus p35S::AtDREB1C-04 and WT versus pRD29A::AtDREB1C-07; **Figure 11**). Compared with WT, 629 unigenes were upregulated and 351 were downregulated in p35S::AtDREB1C-04, while 1,148 unigenes were upregulated and 378 were downregulated in pRD29A::AtDREB1C-07 (Supplementary Table S5). Additionally, compared with p35S::AtDREB1C-04, 342 unigenes were upregulated and 101 were downregulated in pRD29A::AtDREB1C-07 transgenic *S. miltiorrhiza* (Supplementary Table S5).

**FIGURE 12:**
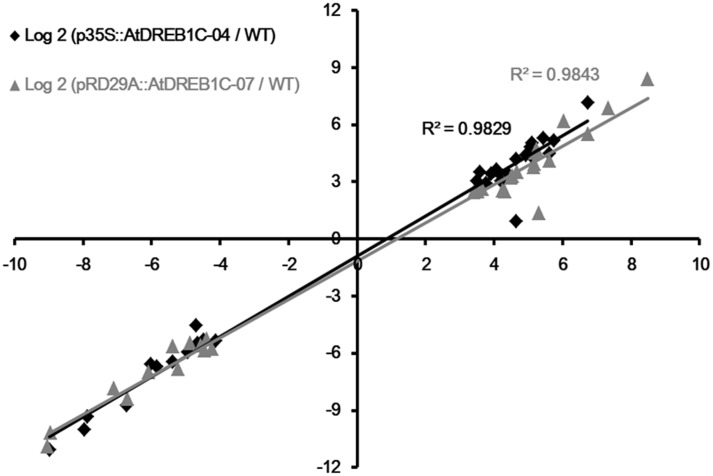
**Correlation of fold changes determined by RNA-Seq (*x*-axis) and qRT-PCR data (*y*-axis).** WT, wild type; p35S::AtDREB1C-04, p35S::AtDREB1C transgenic line 04; pRD29A::AtDREB1C-07, pRD29A::AtDREB1C transgenic line 07.

To validate the results of expression profiling, we selected 40 genes that were significantly upregulated and 20 that were significantly downregulated in the AtDREB1C transgenic plants for further analysis (Supplementary Table S6). Quantitative RT-PCR was performed on a set of 30 genes (18 significantly upregulated genes and 12 significantly downregulated genes) selected at random from among the significant DEGs in the transgenic lines compared to WT plants following drought treatment (Supplementary Table S6). The relative expression levels of these genes were similar to the expression profiles determined from the respective RNA-seq data (Supplementary Figure S1). A high correlation (*R*^2^ > 0.98) was found between the qRT-PCR and RNA-seq results (**Figure 12**), confirming the accuracy of the RNA-seq data.

### COG Enrichment and KEGG Pathway Analysis of DEGs

**FIGURE 13:**
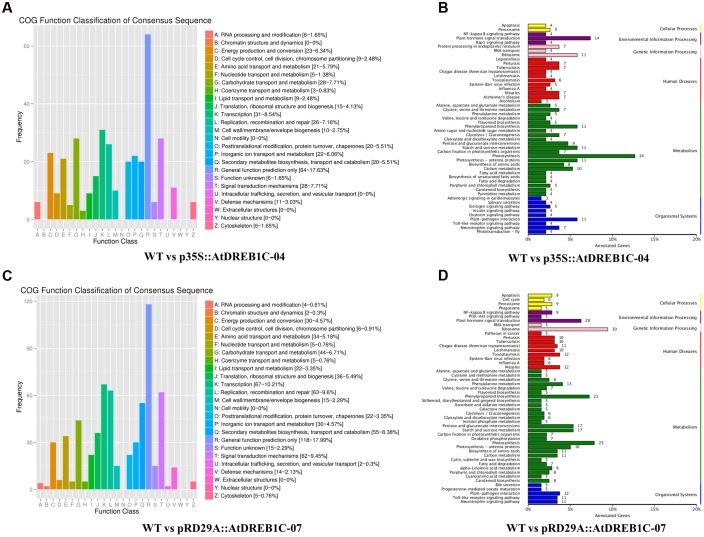
**COG and KEGG classification of DEGs in two comparisons [WT versus p35S::AtDREB1C-04 (A,B)** and WT versus pRD29A::AtDREB1C-07 **(C,D)**]. WT, wild type; p35S::AtDREB1C-04, p35S::AtDREB1C transgenic line 04; pRD29A::AtDREB1C-07, pRD29A::AtDREB1C transgenic line 07.

Annotations of DEGs against the COG database showed that more than 17% of DEGs in both comparisons (WT versus p35S::AtDREB1C-04 and WT versus pRD29A::AtDREB1C-07) could not be annotated accurately and were therefore classified in the cluster ‘general function prediction only.’ Based on the number of annotated genes in each category, ‘transcription’ and ‘signal transduction mechanisms’ were two of the top three categories in the two DEG sets (WT versus p35S::AtDREB1C-04 and WT versus pRD29A::AtDREB1C-07) according to the COG database (**Figures 13A,C**), which suggests that DEGs in these groups play important roles in the drought stress response of AtDREB1C transgenic *S. miltiorrhiza.*

**FIGURE 14:**
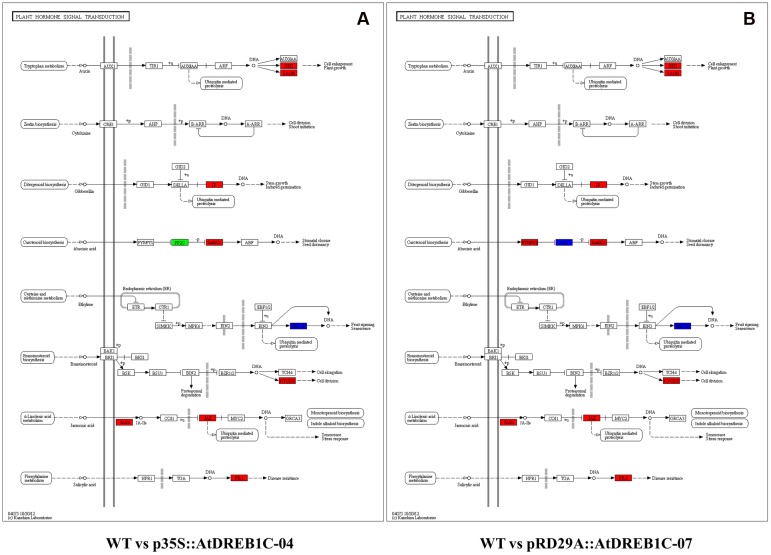
**Analysis of DEGs related to plant hormone signal transduction in two comparisons [WT versus p35S::AtDREB1C-04 (A)** and WT versus pRD29A::AtDREB1C-07 **(B)**]. Red/green indicate up-/downregulated DEGs, respectively. Blue indicates DEGs with mixed patterns of regulation. WT, wild type; p35S::AtDREB1C-04, p35S::AtDREB1C transgenic line 04; pRD29A::AtDREB1C-07, pRD29A::AtDREB1C transgenic line 07.

Furthermore, we analyzed the regulatory pathways of the DEGs using KEGG analysis to obtain gene functional and genomic information. The top 50 enriched KEGG pathways of DEGs among the WT versus p35S::AtDREB1C-04 and WT versus pRD29A::AtDREB1C-07 comparisons are shown in **Figures 13B,D**, respectively. Many DEGs from both comparisons were enriched in the KEGG pathways of photosynthesis, plant hormone signal transduction, ribosome, and phenylpropanoid biosynthesis. Plant hormone regulatory pathways, including auxin, cytokinin, gibberellin, ABA, ethylene, brassinosteroid, jasmonic acid, and salicylic acid pathways (**Figure 14**), ranked second and fourth among the top 50 KEGG pathways of DEGs identified in the WT versus p35S::AtDREB1C-04 and WT versus pRD29A::AtDREB1C-07 comparisons, respectively (**Figures 13B,D**). Except for the cytokinin pathway, other hormone regulatory pathways appear to be involved in AtDREB1C-mediated enhanced drought tolerance in transgenic *S. miltiorrhiza* (**Figure 14**).

Analysis of auxin, gibberellin, brassinosteroid, jasmonic acid, and salicylic acid KEGG pathways showed that all DEGs were upregulated in both p35S::AtDREB1C and pRD29A::AtDREB1C transgenic plants (**Figure 14**). The genes encoding ERF1/2, a pivotal regulator in the ethylene pathway, exhibited mixed expression patterns in both comparisons (WT versus p35S::AtDREB1C-04 and WT versus pRD29A::AtDREB1C-07). The genes for the core components for ABA signaling, PYR/PYL, PP2C, and SnRK2, which function as ABA receptors, repressors, and positive regulators, exhibited various patterns of expression; *PP2C* was downregulated and *SnRK2* was upregulated in the p35S::AtDREB1C transgenic lines, while *PP2C* exhibited mixed patterns of expression, and *PYR/PYL* and *SnRK2* were upregulated in pRD29A::AtDREB1C transgenic *S. miltiorrhiza* (**Figure 14**).

In addition, we analyzed the DEGs between p35S::AtDREB1C-04 and pRD29A::AtDREB1C-07 transgenic lines after drought stress. According to the COG database, more than 15% of DEGs were classified in ‘secondary metabolites biosynthesis, transport and catabolism,’ which was the largest category with accurate annotation (Supplementary Figure S2A). Furthermore, ‘phenylalanine metabolism’ and ‘phenylpropanoid biosynthesis’ were the top two KEGG pathways of DEGs identified in the comparison of p35S::AtDREB1C-04 versus pRD29A::AtDREB1C -07 (Supplementary Figure S2B). Compared with p35S::AtDREB1C-04, most of the significant DEGs were upregulated in pRD29A::AtDREB1C-07. Meanwhile, almost all the significant DEGs were expressed at very low levels in WT (Supplementary Table S7).

## Discussion

Water deficit, a major abiotic stress factor, limits crop yields, thereby affecting the incomes of millions of farmers in arid and semi-arid regions (Ferdous et al., 2015; Todaka et al., 2015; Zhu et al., 2016). Such losses in yields vary greatly depending on the timing, duration, and intensity of the stress. This variability, combined with other location-specific environmental stressors, such as high light and temperature, hamper efforts aimed at producing drought-tolerant plants through conventional breeding (Fang and Xiong, 2015; Thomas, 2015). Compared to traditional breeding and marker-assisted selection, directly introducing stress tolerance-related genes via genetic engineering represents a more effective, rapid approach for increasing abiotic stress tolerance in plants (Patel et al., 2015; Todaka et al., 2015). One important strategy for genetic engineering is to induce a TF gene that regulates several genes involved in abiotic stress (Rehman and Mahmood, 2015). Indeed, overexpressing stress-inducible DREB TF genes activates the expression of many target genes containing DRE elements in their promoters, leading to improved stress tolerance (Morran et al., 2011; Tillett et al., 2012; Guttikonda et al., 2014; Dou et al., 2015; Kidokoro et al., 2015). However, constitutively expressing DREB genes often has various pleiotropic effects on the growth and development of plants, such as growth retardation, dwarfism, and delayed flowering (Sharabi-Schwager et al., 2010; Shavrukov et al., 2015; Yamasaki and Randall, 2016; Wu et al., 2017). Several attempts have been made to overcome the problem of severe growth retardation by reducing the duration of DREB overexpression through the use of stress-inducible promoters (Morran et al., 2011; Datta et al., 2012; Kovalchuk et al., 2013; Ravikumar et al., 2014).

In the present study, we functionally validated the role of AtDREB1C in drought tolerance in *S. miltiorrhiza*. Like the TFs AtDREB1A and AtDREB1B (Wei et al., 2016a,b), ectopic expression of AtDREB1C led to a substantial improvement in the capacity of *S. miltiorrhiza* to survive under severe drought stress conditions, suggesting that these three TFs play similar roles in the plant stress response. When considering the effects of TFs on the growth of transgenic plants, we observed diverse phenotypes among transgenic lines expressing *AtDREB1A*, *AtDREB1B*, and *AtDREB1C* driven by the constitutive CaMV35S promoter: under normal conditions, p35S::AtDREB1A transgenic *S. miltiorrhiza* showed slightly stunted growth (Wei et al., 2016b), p35S::AtDREB1B transgenic lines exhibited normal growth (Wei et al., 2016a), and p35S::AtDREB1C plants exhibited severe dwarfism. Interestingly, no growth inhibition or phenotypic aberrations were observed in transgenic plants harboring any of these genes driven by the stress-inducible RD29A promoter. One possible explanation for these results is that while the results provide no clear evidence for specialized functions for AtDREB1A, AtDREB1B, and AtDREB1C, quantitative differences may exist, with the effects of a TF on a given trait varying among transgenic lines (Gilmour et al., 2004). Meanwhile, the RD29A promoter::DREB1 system has proven to be highly effective for overexpressing DREB1 proteins during exposure to stress (Ravikumar et al., 2014; Ma et al., 2015).

We performed physiological and transcriptome analysis to elucidate the mechanisms involved in the drought resistance of the transgenic plants. Physiological tests suggested that the higher chlorophyll content (**Figure 8B**), photosynthetic capacity (**Figures 9A–C**), and SOD, POD, and CAT activity (**Figures 7B–D**) enhanced the drought stress resistance of transgenic plants compared to WT. Similar effects were observed in other plant species, including tomato (Lee et al., 2003), sugarcane (Reis et al., 2014), and *Lolium perenne* (Li et al., 2011). Transcriptome analysis has been used to elucidate the gene regulatory networks involved in abiotic stress responses in several plant species, such as *A. thaliana* (Mao et al., 2016), rice (Oono et al., 2014), potato (Zhang N. et al., 2014), *Brassica juncea* (Bhardwaj et al., 2015), and *Robinia pseudoacacia* L. ‘Idaho’ (Xiu et al., 2015). Transcriptome analysis of *S. miltiorrhiza* identified a number of DEGs between the AtDREB1C transgenic lines and WT, suggesting that their corresponding proteins play specific roles in the drought stress response. COG analysis indicated that DEGs involved in ‘transcription,’ ‘signal transduction mechanisms,’ ‘carbohydrate transport and metabolism,’ and ‘replication, recombination and repair’ play important roles in drought tolerance (**Figures 13A,C**). The main pathways regulated by AtDREB1C, according to KEGG enrichment analysis, include photosynthesis, plant hormone signal transduction, phenylpropanoid biosynthesis, ribosome, starch and sucrose metabolism, and plant–pathogen interactions (**Figures 13B,D**).

In our previous study, global expression profiling was compared for WT and pRD29A::AtDREB1A-31 transgenic line after drought stress (Wei et al., 2016b). Similarly, numerous genes involved in signal transduction, transcriptional activation, photosynthesis, protein protection, carbohydrate transport and metabolism were significantly upregulated in pRD29A::AtDREB1A-31 plants. Further analysis showed that, the top 10 categories of COG enrichment of DEGs were almost the same between AtDREB1A and AtDREB1C transcriptome. Besides, photosynthesis, plant hormone signal transduction, ribosome, and plant–pathogen interactions were also the main pathways regulated by AtDREB1A TF. Furthermore, qRT-PCR analysis revealed that the expression of 30 upregulated genes, which selected from AtDREB1A and AtDREB1C transcriptome, were increased significantly in AtDREB1B transgenic plants (Wei et al., 2016a; Wei et al., unpublished), indicating that AtDREB1-type TFs may regulate the same downstream genes under drought stress (Gilmour et al., 2004).

Phytohormones are major regulators of plant development, growth, and physiological responses, and they mediate environmental stress responses by integrating environmental stimuli and regulatory networks (Jaillais and Chory, 2010; Depuydt and Hardtke, 2011; Xiu et al., 2015). Manipulating plant hormone metabolism and signaling processes represents a promising strategy for increasing plant stress tolerance (Cabello et al., 2014). ABA is a key regulator of plant responses to environmental stress, particularly osmotic stress (Seiler et al., 2011; Jones, 2016). The essential components of ABA signaling have recently been identified, and their mode of action has been clarified. In the current model, ABA signaling involves three core components: receptors (PYR/PYL/RCAR), protein kinases (SnRK2/OST1), and protein phosphatases (PP2C), (Cramer et al., 2011; Osakabe et al., 2014; Kong et al., 2015; Chen et al., 2016). Overexpressing *FpDREB2A* in *R. pseudoacacia* increases drought stress resistance. Analysis of the root transcriptome of *FpDREB2A* transgenic line revealed that PYL, PP2C, and SnRK2 expressed in an interval of down- and up-regulation, which suggested that the transgenic plants regulated ABA signal transduction and supposed to be involve in drought stress responses (Xiu et al., 2015). In this study, we found that ABA signaling pathways were altered in transgenic *S. miltiorrhiza* compared to WT (**Figure 14**). The up- or down-regulation of the core components of ABA signaling in the transgenic plants may give the plants the flexibility needed to adapt to drought stress, although the exact mechanisms remain unclear.

Auxin also plays a critical role in plant drought stress responses (Depuydt and Hardtke, 2011). The expression levels of numerous auxin-related genes are altered under dehydration stress (Jain and Khurana, 2009). The induction of *YUCCA7 (YUC7)*, encoding a flavin monooxygenase that functions in the tryptophan-dependent auxin biosynthetic pathway, increases endogenous auxin levels and enhances drought resistance in Arabidopsis (Du et al., 2012; Lee and Luan, 2012). Auxin alters the expression of numerous genes, including primary auxin-responsive genes of the Aux/IAA, GH3, and SAUR gene families. Aux/IAA gene family members are implicated in the light-induced regulation of auxin responses. Although to date, no SAUR genes have been functionally characterized, SAUR proteins bind to calcium/calmodulin, suggesting that they play a role in auxin signaling via calcium ions (Park et al., 2007). GH3s, which are members of the acyl adenylate-forming firefly luciferase superfamily, catalyze the adenylation of specific substrates (Wang et al., 2008). GH3-mediated auxin homeostasis is an essential component of the complex auxin regulatory network underlying stress adaptation in Arabidopsis (Park et al., 2007). In the present study, we identified upregulated DEGs in the auxin pathway, including GH3 and SAUR family members (**Figure 14**), suggesting that these two groups of auxin-responsive genes play specific roles in AtDREB1C-mediated drought tolerance in *S. miltiorrhiza*.

Other hormones, including gibberellin, ethylene, brassinosteroid, jasmonic acid, and salicylic acid, also play direct or indirect roles in the abiotic stress response (Peleg and Blumwald, 2011; Cabello et al., 2014; Zhou et al., 2014; Chen et al., 2016). For example, ethylene may modulate the expression of genes considered to be effectors of ethylene signaling (Shi et al., 2012). Environmental stress induces ethylene accumulation, which increases plant survival under adverse conditions. Recent evidence indicates that phytohormones function in multiple processes. Crosstalk between various plant hormone regulatory pathways results in synergetic or antagonistic interactions, which play crucial roles in abiotic stress responses (Depuydt and Hardtke, 2011; Peleg and Blumwald, 2011; Song et al., 2014; Shi et al., 2015; Chen et al., 2016). Plant growth retardation often occurs when plant hormone homeostasis is altered. Excess ABA biosynthesis may deplete the pool of precursors needed for chlorophyll biosynthesis, thereby leading to growth retardation (Sreenivasulu et al., 2012). GH3-8 is an IAA–amido synthetase that alters auxin homeostasis; overexpressing *GH3-8* leads to abnormal plant morphology, as well as retarded growth and development, in transgenic rice (Ding et al., 2008). Therefore, we speculate that constitutive expression of AtDREB1C alters plant hormone homeostasis, especially at the vegetative stage, which might be the main cause of the dwarf phenotypes of the p35S::AtDREB1C transgenic lines.

Taken together, our findings indicate that AtDREB1C plays important roles in the drought resistance of transgenic *S. miltiorrhiza.* The improved drought tolerance of these plants was associated with improved photosynthesis and antioxidant defense systems. Analysis of the transcriptome of AtDREB1C transgenic plants showed changes in the expression of genes involved in pathways including photosynthesis, plant hormone signal transduction, phenylpropanoid biosynthesis, ribosome, starch and sucrose metabolism, and plant–pathogen interactions. In particular, the differential expression of genes involved in plant hormone signaling are thought to be one of the main causes of increased drought tolerance in the AtDREB1C transgenic plants. Further investigation of the effects of the AtDREB1C TF on the crosstalk between different plant hormones, including biosynthesis and signaling, as well as their roles in plant growth and responses to abiotic stresses, will help elucidate the mechanism underlying the effects of AtDREB1C-mediated plant hormone homeostasis on plant growth and stress responses.

## Author Contributions

TW, KD, CC, and YZ designed the experiments, analyzed the data and wrote the manuscript. TW and KD performed the main experiments in this study. QZ, YG, and YL contributed reagents, materials, and helped in the stress experiments. QZ, YG, MY, and LZ helped with the detection of photosynthesis and enzyme activities. XZ, CW, and ZL contributed to data analyses and discussion. All authors read and approved the final manuscript.

## Conflict of Interest Statement

The authors declare that the research was conducted in the absence of any commercial or financial relationships that could be construed as a potential conflict of interest.
