# Supplementary material for: Modulating AtDREB1C Expression Improves Drought Tolerance in Salvia miltiorrhiza
Source: Front Plant Sci. 2017 Jan 24;8:52. doi: 10.3389/fpls.2017.00052 (PMC5259653; doi:10.3389/fpls.2017.00052)
Supplement: Supplementary file 1 [file Data_Sheet_1.pdf]

Table S1 Oligo used in this study.

Oligo name	Sequence (5’ to 3’)	Oligo name	Sequence (5’ to 3’)	Oligo name	Sequence (5’ to 3’)
AtDREB1C-F	GCCCATGGTCACTCCAACATTTCTAT	RD29A-F	GGATCCTTTATTTCTTCGACTC	GUS-F	GTCGCGCAAGACTGTAACCA
AtDREB1C-R	GGGGTCACCCATTCGTTTCTCACAAC	RD29A-R	CCATGGTCCAATAGAAGTAATC	GUS-R	CGGCGAAATTCCATACCTG
35S-F	AACAGAACTCGCCGTAAAG	Actin-F	GGTGCCCTGAGGTCCTGTT	18S-F	CCAGGTCCAGACATAGTAAG
35S-R	TAGTGGGATTGTGCGTCAT	Actin-R	AGGAACCACCGATCCAGACA	18S-R	GTACAAAGGGCAGGGACGTA
**Primers for RNA-seq data validation**					
c36299-F	AGTCCACTCCAGCAACAGGC	c27900-F	CGGGATCGGCCAAATGA	c26640-F	GGGGTCCACCACTAACACTGAAT
c36299-R	TCATTCACCCACAACTCCACC	c27900-R	GGCTTCGGCAAGGATGAGA	c26640-R	TGATGGGTCGTCAAACCTTCC
c36313-F	GTAAGGCATTGTGCTTGGTGG	c27305-F	GCCATCTTCGTCCTCTTGTGC	c33925-F	CGTCCACCAGGCTCTTCAGG
c36313-R	GCTTCATGGCTTCGTTGTCG	c27305-R	CCGTTGTTGGGATTGCTGTG	c33925-R	CCCCCATTAGCAAGGTCCAA
c21366-F	CCTATGTCATCGGAGGGTCAGT	c27788-F	AATCCTGGCGATGGACGAAT	c36270-F	GAGATCCCGACCTGAACCTGT
c21366-R	ACACAGGAGTCTCATCCACCAAG	c27788-R	CGGCGGAGATGTACTGACCC	c36270-R	AACTGCCCGCATCAACTACC
c24745-F	ACATTGGCTTTGATTGCGGT	c32477-F	TCGTGCCCGTGCCTTTC	c36450-F	TGACGATGACGACGCTGATGT
c24745-R	TGCTAACTTGCTCAGCGGATT	c32477-R	CCGCCGCCAGATTGACTT	c36450-R	GGCACGGGATGAGTTCTTGATAC
c25147-F	CCAAAGGAGCATGGAGAAGG	c21729-F	CCCCTTCCACCTGTCTTCTTTC	c8174-F	CCCAAACAGAGCCTGAATCCC
c25147-R	GCACGCAGAATCCCACAAA	c21729-R	TGCCGACTTCGTCATTCCTG	c8174-R	TCACCGAAACCCTCCTCATCC
c15204-F	AATAGAGAATGGTCCGTGCCG	c15651-F	CGTGGCGAAGGGCATCA	c36274-F	ACACCACCTCGTAGCCCTCAG
c15204-R	TCCTCGTATTCATGGGAGTTGC	c15651-R	GGAACACCCAGCCGTAGAAGT	c36274-R	CCAAACGCATCGTCATCAAAG
c36306-F	GGCGGTTCGTTGGTGGTTAG	c28656-F	ATCAGACGGGCGTCACCAAC	c21381-F	TATGAGCCACGACGGACGAT
c36306-R	ATTGCGACGGTGGATGCTG	c28656-R	ACCATGCGGAAGTCGAGGG	c21381-R	CGCCACCCAGATATTGAACG
c15322-F	CCTTTTCCTCCTGAGGCTTGT	c22292-F	CGCATAGGCTTGGAAGGGTC	c36492-F	TGCTCGGAGGTGTAGATTCGG
c15322-R	CCTTGGGCACTTCCACTTTG	c22292-R	TGCTGAAGGTGGCTCACGAAT	c36492-R	CCAAACAGTTCCTGAGGGTGC
c21655-F	GCCGATTCCTCATCCTCTTCA	c28751-F	GCCTGGCACAAGAGCAACCT	c36620-F	ACCTGTCGGAATCGGCACTT
c21655-R	CATTCGGGCTGAGCAACAAC	c28751-R	GAATGACCCTCGCCGTAATCC	c36620-R	CCGCCTGTATGGCTGTCTTG
c8013-F	TCCACCAGTTCCACTTTGCC	c28750-F	TACCCTTCGCATTTCCCTCC	c32894-F	AGTTTCAGCATACGGGATACACC
c8013-R	CGCCGTTTCAGCCCTACA	c28750-R	CCGCTACGCCTCCAACCT	c32894-R	ACCTCCCAAACTTTCCACTGC

Table S2 Statistics of sequencing data.

Samples	Read Number	Base Number	GC Content	%≥Q30
WT	18,810,158	5,672,604,656	50.16%	85.32%
p35S::AtDREB1C-04	17,445,426	5,264,372,548	48.50%	90.02%
pRD29A::AtDREB1C-07	18,966,357	5,717,700,962	49.77%	90.58%

Table S3 Assembling results for the *Salvia miltiorrhiza* transcriptome.

Length Range	Contig	Transcript	Unigene
200-300 nt	13,104,929(99.63%)	38,147(23.57%)	32,431(43.98%)
300-500 nt	20,329(0.15%)	25,418(15.71%)	16,791(22.77%)
500-1000 nt	14,160(0.11%)	29,932(18.49%)	11,461(15.54%)
1000-2000 nt	9,322(0.07%)	39,250(24.25%)	8,115(11.00%)
≥2000 nt	4,749(0.04%)	29,097(17.98%)	4,950(6.71%)
N50 Length	41	1,912	1,182
Mean Length	40.63	1147.32	657.67
Total Number	13,153,489	161,844	73,748
Total Length	534,391,591	185,686,058	48,502,152

Table S4 Summary of Unigene annotations.

Databases	Annotated sequence	300≤length<1000	length≥1000
COG	14182	4955	5090
GO	26570	9944	7109
KEGG	19338	7191	5235
KOG	24965	8782	7812
Pfam	28874	10648	10944
Swiss-Prot	29142	11169	9221
NR	38478	15114	9309
Total	43428	16182	12301

Table S5 Statistics of different expression genes (DEGs).

DEG Set	All DEGs	Up regulated	Down regulated
WT vs p35S::AtDREB1C-04	980	629	351
WT vs pRD29A::AtDREB1C-07	1526	1148	378
p35S::AtDREB1C-04 vs pRD29A::AtDREB1C-07	443	342	101

Table S6 Significant DEGs in AtDREB1C transgenic lines compared to WT after drought treatment.

S.miltiorrhiza unigene ID	RefSeq ID	Description	WT	p35S::AtDREB1C-04	pRD29A::AtDREB1C-07	$Log2FC_1$	$Log2FC_2$
c36380	EF523038.1	AtDREB1C	0	163.20	50.53	11.92	10.30
c18148	CAA35093	Phosphinothricin acetyl transferase (Bar)	1.14	827.02	1313.02	9.72	10.24
**Significantly up regulated genes**							
c17887	XP_012837696.1	Apyrase-like	0.16	101.68	97.77	9.29	9.18
c36299	XP_011092473.1	Pathogenesis-related leaf protein 6-like	0.64	66.55	234.12	6.74	8.49
c36313	XP_012486166.1	Putative lipid-transfer protein DIR1	2.0	101.51	316.07	5.76	7.33
c35078	XP_011085796.1	Metalloendoproteinase 1-like	1.43	76.47	196.25	5.87	7.16
c21366	XP_002326087.2	Xyloglucan endotransglucosylase	0.88	40.50	92.18	5.63	6.75
c18325	XP_012852687.1	Aquaporin TIP2-1-like	1.32	68.78	102.59	5.82	6.33
c21659	XP_011071173.1	Photosystem II protein psbY-2	3.24	176.91	216.60	5.92	6.14
c24745	XP_011070106.1	Proline-rich protein	3.39	133.28	213.23	5.43	6.04
c18932	AAM21199.1	Pathogenesis-related protein 5-1	1.13	179.53	71.78	7.41	6.03
c17927	XP_011007955.1	Chlorophyll a-b binding protein CP24 10A	4.14	121.57	253.38	5.02	6.01
c21509	XP_011072947.1	Zinc finger protein CONSTANS-LIKE 16	1.04	59.49	63.95	5.96	6.0
c24907	ABU87404.1	SMLII	2.74	50.0	156.76	4.33	5.91
c36375	XM_013731549.1	Extensin-like	1.86	65.71	95.40	5.18	5.66
c25147	XP_011077002.1	Chlorophyll a-b binding protein 6A	20.05	621.07	922.51	5.12	5.62
c15204	XP_011080876.1	Cation/H(+) antiporter 18	5.19	114.62	190.48	4.66	5.32
c18208	XP_011094690.1	Peroxidase 21	1.63	62.11	61.29	5.38	5.29
c14610	XP_003597750.1	Late embryogenesis abundant protein	1.46	34.26	54.39	4.68	5.28
c36306	XP_011090031.1	Photosystem II 5 kDa protein	4.48	137.32	162.33	5.08	5.25
c15322	XP_011088934.1	Photosystem I subunit O	8.01	219.03	275.67	4.93	5.19
c21655	XP_011079994.1	Photosystem II reaction center W protein	11.40	347.48	388.07	5.09	5.18
c28459	XP_012836431.1	Aspartic proteinase nepenthesin-1	11.12	180.06	374.62	4.18	5.17
c17877	XP_011087853.1	Chlorophyll a-b binding protein CP24 10A	4.66	58.14	161.86	3.76	5.17
c8013	XM_010039399.1	Chlorophyll a-b binding protein CP29.2	19.30	431.93	645.91	4.65	5.16
c17715	XM_011084787.1	Photosystem I reaction center subunit IV	6.86	126.34	199.06	4.35	4.93
c15317	XM_011098218.1	Chlorophyll a-b binding protein 91R	11.97	96.12	333.10	3.01	4.89
c32058	XM_006658205.1	Oxygen-evolving enhancer protein 2	21.17	490.28	599.16	4.70	4.82
c27900	XM_008381819.1	Chlorophyll a-b binding protein 8	22.90	359.63	544.43	4.14	4.67
c18358	XM_010036974.1	Photosystem I reaction center subunit III	10.78	149.91	239.02	3.96	4.56
c27305	XP_011077566.1	Vacuolar cation/proton exchanger 3	3.54	67.47	77.99	4.41	4.55
c27788	XP_012084444.1	Probable fructose-bisphosphate aldolase 2	27.73	375.41	581.62	3.93	4.49
c18381	XP_004244001.1	Photosystem I reaction center subunit V	18.92	219.17	379.31	3.70	4.42
c32477	XP_009759927.1	Oxygen-evolving enhancer protein 1	51.23	626.20	955.20	3.78	4.32
c21729	XP_011076800.1	Rubisco activase 1	146.99	2428.86	2635.05	4.21	4.26
c15134	XP_012852677.1	Photosystem I reaction center subunit VI	25.03	384.99	417.42	4.11	4.15
c18397	XP_011074720.1	Plastocyanin-like	23.80	317.18	357.89	3.90	4.0
c15139	XP_011083089.1	Photosystem I reaction center subunit IV A	25.34	367.57	357.54	4.02	3.91
c26960	XP_002303455.1	Phosphoribulokinase family protein	6.77	99.06	90.87	4.03	3.84
c15651	XP_011071704.1	Geranylgeranyl diphosphate reductase	7.24	78.59	86.25	3.60	3.67
c28656	XP_011090165.1	Serine-glyoxylate aminotransferase	23.07	344.53	248.41	4.07	3.52
c22292	XP_011090789.1	Flavanone 3-dioxygenase	6.10	61.57	60.75	3.50	3.41
**Significantly down regulated genes**							
c17196	XP_013464786.1	Wound-induced protein	59.15	1.61	5.64	-4.97	-3.29
c14184	XP_011092508.1	Zinc finger protein AZF2	93.75	7.09	7.14	-3.73	-3.61
c27906	XP_011095949.1	DOMON domain-containing protein	408.78	16.19	31.22	-4.49	-3.61
c36272	XP_011080767.1	Alanine-glyoxylate aminotransferase 2	777.74	39.61	45.24	-4.13	-4.01
c28751	XP_011070248.1	Expansin-like B1	1148.82	24.62	56.83	-5.37	-4.24
c28750	XP_006385562.1	Expansin-related protein 1 precursor	727.12	21.01	32.39	-4.94	-4.39
c26640	XP_011082692.1	Early nodulin-like protein 2	809.99	41.14	34.42	-4.13	-4.46
c33925	XP_012847718.1	ABC transporter G family member 21	81.83	3.25	3.21	-4.47	-4.57
c36270	AAN05097.1	Lectin	867.94	30.87	27.73	-4.64	-4.87
c36450	XP_011076012.1	Probable protein phosphatase 2C	135.08	1.82	3.34	-6.02	-5.23
c8174	EYU40157.1	Hypothetical protein MIMGU	216.90	7.35	4.75	-4.69	-5.38
c34446	XP_011084988.1	Heat shock protein-like	495.30	29.93	10.02	-3.88	-5.52
c18796	XP_011096322.1	Desiccation-related protein PCC3-06	184.86	3.67	3.14	-5.47	-5.76
c36274	XP_011085010.1	Delta-1-pyrroline-5-carboxylate synthase	922.63	14.29	12.78	-5.84	-6.08
c21381	XP_013454869.1	Phosphatidylethanolamine-binding protein	80.74	0.62	0.67	-6.73	-6.70
c36492	AJD25216.1	Cytochrome P450 CYP92B29	78.52	0.27	0.51	-7.86	--7.10
c18763	XP_011099725.1	NAC transcription factor 25-like	77.89	0.38	0.14	-7.35	-8.63
c36615	XP_011073727.1	Uncharacterized protein	74.72	0	0.09	-10.15	-8.89
c36620	XP_011095083.1	Aldo/keto reductase	85.12	0.27	0.12	-7.95	-8.95
c32894	XP_012834490.1	MLO-like protein 6	261.58	0.44	0.45	-8.97	-9.03

Table S7 Significant DEGs between p35S::AtDREB1C-04 and pRD29A::AtDREB1C-07 transgenic lines after drought stress.

S.miltiorrhiza unigene ID	RefSeq ID	Description	WT	p35S::AtDREB1C-04	pRD29A::AtDREB1C-07	Log2FC
**Significantly up regulated genes**						
c35069	XP_011102248.1	uncharacterized protein LOC105180271	0	0.37	37.22	6.47
c31130	XP_012829322.1	Sieve element	1.54	0.87	50.47	5.76
c35152	XP_011099650.1	Germin-like protein subfamily 1 member 13	0.82	1.17	52.45	5.36
c35375	XP_012828920.1	Hyoscyamine 6-dioxygenase	0.12	1.27	42.33	4.95
c8601	XP_012856133.1	Probable 2-oxoglutarate-dependent dioxygenase AOP1	0.06	1.90	61.54	4.93
c31586	NP_175689.1	2OG-Fe(II) oxygenase superfamily	0.13	1.02	22.33	4.34
c27931	XP_011092713.1	Probable carbohydrate esterase	0	1.12	26.01	4.31
c23712	EYU29679.1	hypothetical protein MIMGU_mgv1a017470mg	0	1.34	28.63	4.27
c11119	XP_012844424.1	Dirigent-like protein	0.23	3.06	61.11	4.22
c34691	XP_011082333.1	uncharacterized protein LOC105165136	0.24	1.10	21.33	4.19
c25673	XP_011080630.1	Aquaporin TIP2-1	1.13	1.02	20.07	4.15
c29789	XP_011083298.1	Polyphenol oxidase I	0.36	1.63	21.80	3.64
c23329	NP_001318059.1	Polyphenol oxidase F	0.72	1.90	24.12	3.54
c26385	XP_011101276.1	High-affinity nitrate transporter 3.1	0.18	1.80	21.24	3.46
c26864	XP_015079001.1	Tetrahydrocannabinolic acid synthase	3.38	3.01	34.37	3.44
c33283	XP_011070229.1	Protein NRT1/ PTR FAMILY 7.3	3.67	11.44	126.72	3.40
c31581	XP_009785374.1	Chalcone and stilbene synthases	0.53	1.99	22.13	3.39
c23391	XP_011097243.1	Papain family cysteine protease	1.39	2.82	30.07	3.33
c33825	XP_011099651.1	Germin-like protein subfamily 1 member 13	1.24	10.53	105.95	3.25
c37045	XP_011094266.1	secoisolariciresinol dehydrogenase-like isoform X1	0.82	2.20	21.81	3.22
C24758	XP_011069614.1	pathogen-related protein	0.81	2.57	24.31	3.15
c27768	XM_011095684.1	Extensin-like protein	0	3.34	32.38	3.12
c32773	XP_009761509.1	Deacetoxyvindoline 4-hydroxylase	19.88	18.23	158.22	3.04
c15685	ABU87404.1	Legume lectin	0.31	6.70	58.52	3.03
**Significantly down regulated genes**						
c22092	XP_011088991.1	uncharacterized protein	3.26	35.25	8.36	-2.14
c27855	XP_011071701.1	Uncharacterized acetyltransferase	1.29	24.85	5.64	-2.21
c15026	XP_007216872.1	hypothetical protein PRUPE_ppa021261mg	3.47	127.71	28.28	-2.24
c31839	XP_011100796.1	Beta-D-xylosidase 1	5.10	233.55	43.88	-2.48
c15805	XP_006340812.1	stress-induced protein KIN2-like	2.61	111.83	19.39	-2.59
c18636	XP_011083572.1	Chaperone protein dnaJ 11	2.78	124.71	21.01	-2.64
c17855	XP_012828897.1	uncharacterized protein LOC105950126	6.90	333.77	42.83	-3.05

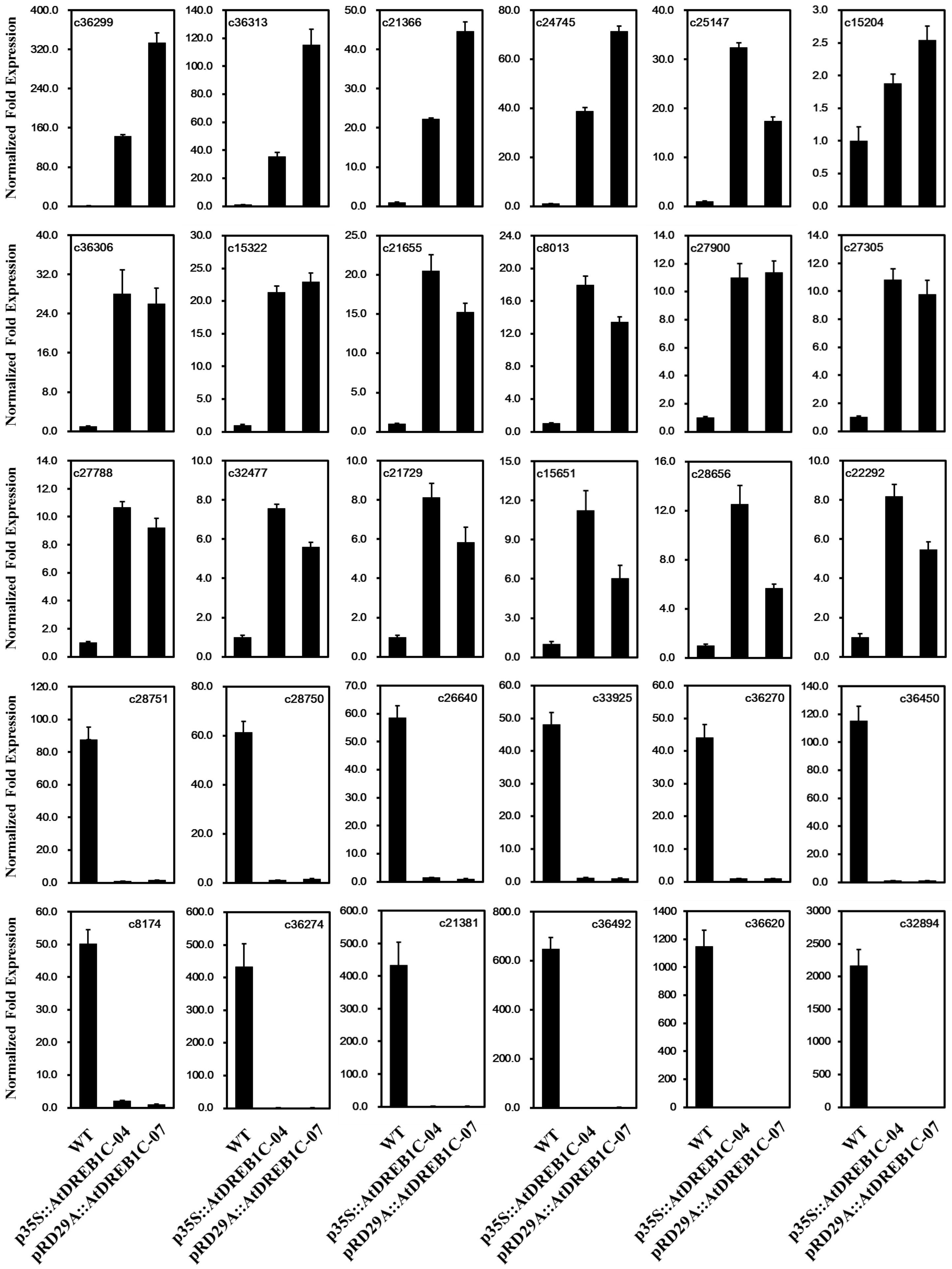

Figure S1 Validation of the RNA-seq results by qRT-PCR.

A

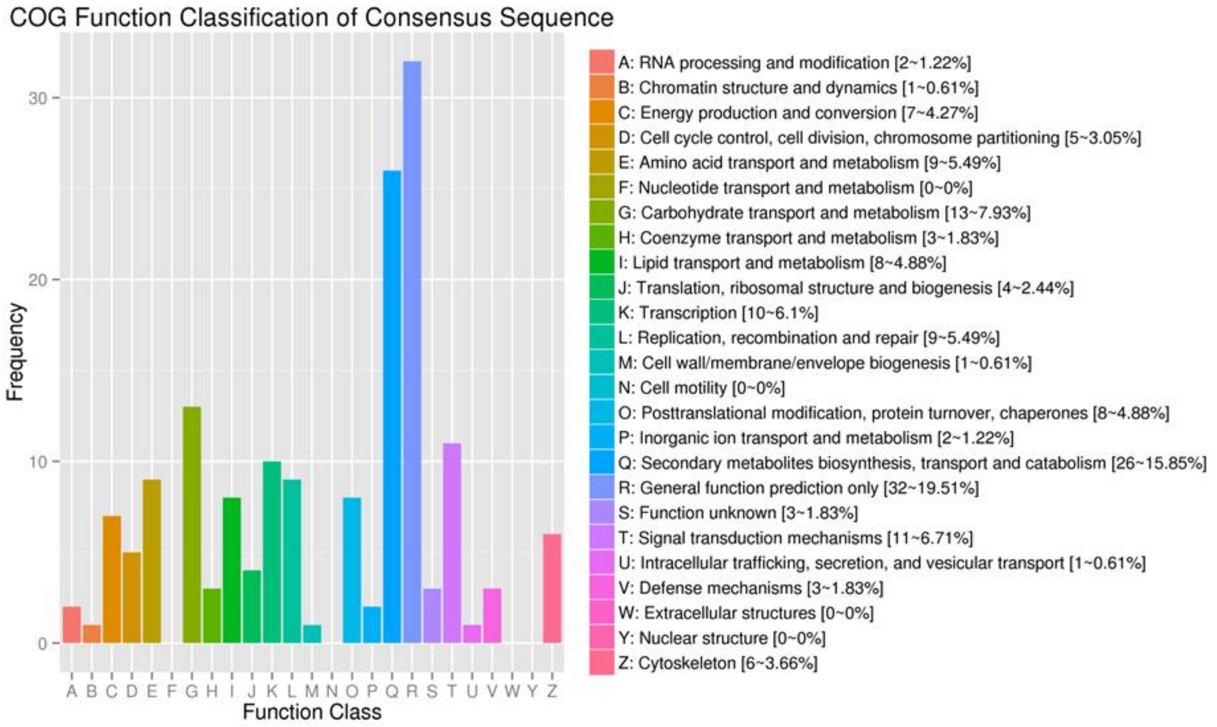

p35S::AtDREB1C-04 vs pRD29A::AtDREB1C-07

B

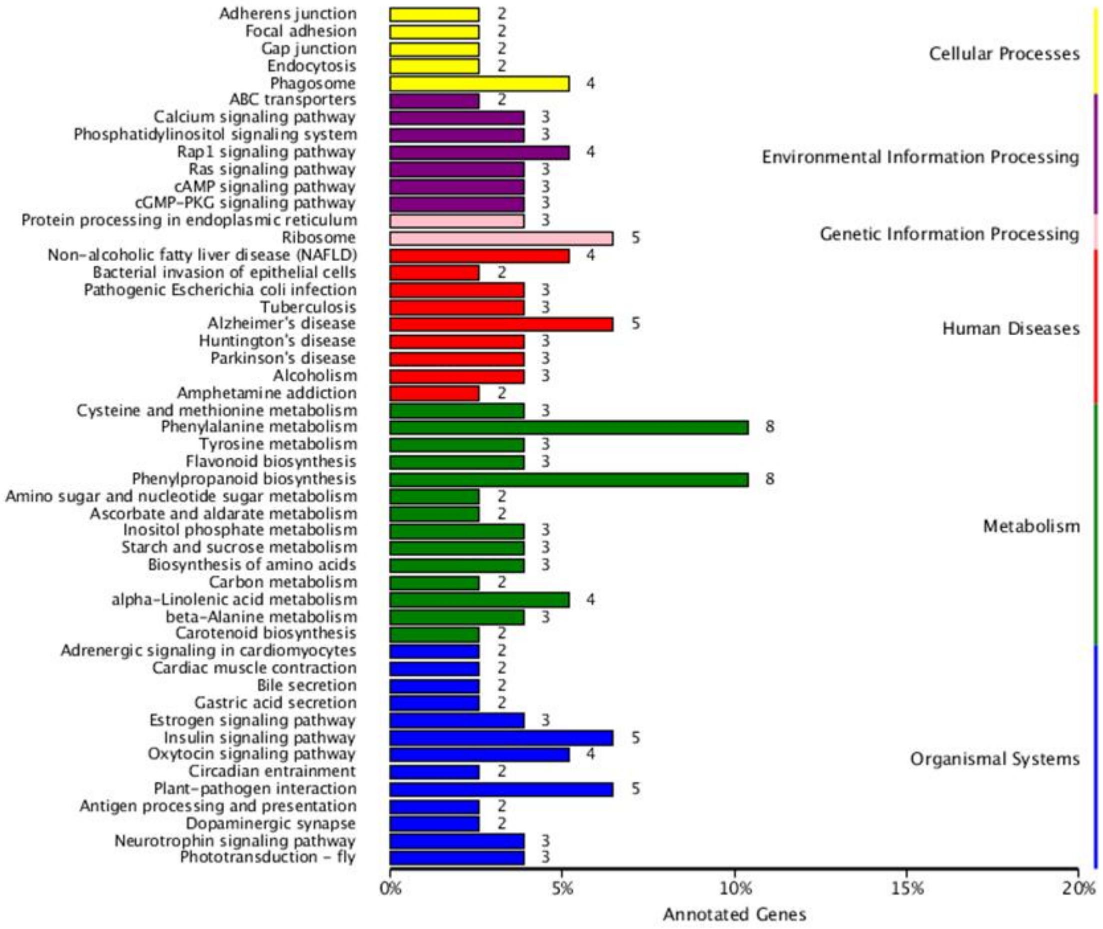

p35S::AtDREB1C-04 vs pRD29A::AtDREB1C-07

Figure S2 COG and KEGG classification of DEGs between p35S::AtDREB1C-04 and pRD29A::AtDREB1C-07 transgenic *Salvia miltiorrhiza* plants after drought stress.
